# Supplementary material for: Elevated Bile Acid 3β,5α,6β-Trihydroxycholanoyl Glycine in a Subset of Adult Ataxias Including Niemann–Pick Type C
Source: Antioxidants (Basel). 2024 May 2;13(5):561. doi: 10.3390/antiox13050561 (PMC11117656; doi:10.3390/antiox13050561)
Supplement: Supplementary file 1 [file antioxidants-13-00561-s001.zip › antioxidants-2844844-supplementary.pdf]

Supplementary materials

Table S1. Complete list of numbers allocated to the 184 participants in the study with plasma concentrations in nmol/L of 3β,5α,6β-trihydroxycholanoyl glycine (3β,5α,6β -triOH-gly) and 3β,7β-trihydroxy-5-cholenoyl glycine (3β,7β-diOH-Δ5-gly). *Plasma sample 20 is the one from the individual with NPC.

Plasma ID number	3β,7β-diOH-Δ5-Gly	3β,5α,6β-triOH-Gly	ID	3β,7β-diOH-Δ5-Gly	3β,5α,6β-triOH-Gly	ID	3β,7β-diOH-Δ5-Gly	3β,5α,6β-triOH-Gly	ID	3β,7β-diOH-Δ5-Gly	3β,5α,6β-triOH-Gly	ID	3β,7β-diOH-Δ5-Gly	3β,5α,6β-triOH-Gly
1	10.7	8.4	41	108.1	9.6	83	136	29.3	129	100.4	21.9	172	87.8	37.5
2	79.4	8.9	42	35.6	14.2	85	60.6	27.2	130	61.6	18.3	173	133.6	36.2
3	236.9	15.7	43	162.5	10.8	86	45.9	57.8	131	284.1	38.7	174	172.5	32.9
4	185.3	38.6	44	61.9	44.9	87	84.3	69.6	132	109.6	28.1	175	400.6	101
5	10.1	7.4	46	116.2	41.2	88	58.5	111.5	134	26.3	28.4	176	41.9	60.1
6	117.9	6.9	47	31	14.1	89	127.1	21	135	18.5	20.2	177	110.7	35.7
7	120.8	20	48	36.1	10.2	90	35.5	22.6	136	250	34.9	178	26.5	29.9
8	114	7.2	49	112.1	22.7	91	82	36.5	137	46.2	22	179	272.4	42.2
9	45.4	14	50	37.1	14.4	92	44.2	63.2	138	19.6	19.7	181	499.7	30.7
10	188.9	36.7	51	450	108.8	93	47.5	21.3	139	39.4	24.4	182	95.8	29.9
11	75.5	12.8	52	42.4	85.2	94	67.1	28.9	140	38.4	33.3	183	174.1	35.7
12	11.8	10.8	53	75.8	36	95	277.4	18.3	141	21.1	19.7	184	115.7	44.4
13	60.9	59	54	71.2	10.9	96	74.7	22.4	142	179.5	18.5	185	964.5	99.7
14	119.9	16.9	55	131.4	10.8	97	39.6	21.7	143	480.1	11.6	186	48.1	50.1
15	64.4	12.4	56	92.4	10.8	98	93.2	23.6	144	4.4	10.3	187	35.1	28.2
16	26.4	8.9	57	386.4	15.6	100	82.6	165.3	145	161.1	12.2	188	697	50.1
17	81.7	11.4	58	108.7	13.1	103	32.3	12.3	146	15.2	20.5	189	97.9	75.9
18	216.6	45.4	60	58.8	16.4	104	45.2	12.2	148	91.6	20.2	190	136.3	33.9
19	95.2	13.9	61	44.2	13.5	105	93.6	15	149	9.8	66.3	191	289.4	67.1
**20***	**78.4**	**123.9**	62	32.4	45.9	106	85.8	35	150	50.7	7.9	192	150.3	38

21	278.5	15.7	63	63	15.9	107	135	19.1	151	92.7	15.6	193	142	37
22	130.4	11.5	64	19	17.8	108	34.1	18.3	152	41.4	6.7	194	108.9	27.9
23	40.7	6.7	65	78.7	17.7	110	249.7	41.1	153	22.3	11.6	196	150.2	43.2
24	46.8	24	66	27.6	12.8	111	24.7	17.6	154	126.6	219.2	197	14	15.5
25	168.3	8.9	67	24.9	38.7	112	417.4	26.3	155	104.9	159.4			
26	23	7.9	68	82.7	17.3	113	21	12.6	156	97.8	43.1			
27	5.7	11	69	24.1	9.5	114	278	36.3	158	141.4	14.7			
28	70.9	12	70	65	34.6	115	113.6	36.7	159	10	4.8			
29	10.2	6.4	71	110.2	12.6	116	51.7	47.6	160	265.3	48.3			
30	375.7	14.6	72	127.5	26.3	117	14	14.1	161	33.9	14.5			
31	93.9	27.8	73	30.6	23.3	118	88.9	21.9	162	108.8	2.3			
32	61.4	28.8	74	43	22.1	119	76.3	24.2	163	89.4	12.9			
33	24.5	8.3	75	280.5	42.2	120	141.3	36.2	164	51.9	11.5			
34	51.2	9.1	76	38.7	23	121	131.6	26.5	165	183.6	17.6			
35	19.3	6.4	77	109.9	20.9	122	13.3	17.3	166	25.3	31.2			
36	260.9	31.1	78	217.6	24.3	123	144.1	38.8	167	122	31.2			
37	63.8	6.1	79	114.2	20.8	124	32.9	32.7	168	104.4	33.6			
38	302.7	52.2	80	21.3	19.2	125	147.7	28.6	169	334	52.5			
39	91.9	11.1	81	217.6	34.8	126	94.2	29.6	170	187.6	36.7			
40	109.5	54.2	82	66.9	22.4	127	204.1	32.1	171	31.6	72.7			

Table S2: The features of patients with an identified genetic diagnosis in our study

Plasma Sample ID	Sex	Birth year	Age at sampling	Genetic Diagnosis	Gene	Comment	3β,7β-diOH-Δ5-Gly	3β,5α,6β-triOH-Gly
1	Male	1957	59	HSP	SPG7	Homozygous for the c.1529C>T p.(Ala510Val) pathogenic variant	10.7	8.4
2	Male	1959	57	HSP	SPG7	Compound heterozygous for c.1454_1462del p.(Arg485_Glu487del) & c.1672A>T p.(Lys558*)	79.4	8.9
18	Male	1966	50	SCA13	KCNC3	Heterozygous for the c.1259G>A p.(Arg420His) pathogenic variant	216.6	45.4
20	Male	1996	20	NPC/primary ciliary dyskinesia	NPC1 & CCDC114	Homozygous for the c.3493G>A p.(Val1165Met) likely pathogenic variant in NPC1 and homozygous for the c.287del p.(Lys96Argfs*23) likely pathogenic variant in CCDC114	78.4	**123.9***
33	Male	1964	52	HSP	SPG7	Compound heterozygous for c.1529C>T p.(Ala510Val) & c.1672A>T p.(Lys558*)	24.5	8.3
47	Male	1955	61	SCA11	TTBK2	Heterozygous for the c.1297_1304del p.(Pro433Argfs*15) likely pathogenic variant	31	14.1
69	Female	1984	32	EA1	CACNA1A	Heterozygous for the c.2042_2043delAG p.(Gln681Argfs*100)	24.1	9.5
75	Female	1983	34	AOA2	SETX	Homozygous presence of c.4161_4162insTT; p. Val1388LeufsTer27	280.5	42.2
78	Female	1959	57	SCA10	ANO10	Homozygous for the c.132dup p.(Asp45Argfs) pathogenic variant	217.6	24.3

81	Female	1945	71	Autosomal dominant or recessive spinocerebellar ataxia (ADSCA/ARSCA)	ANO10	Homozygous for the c.132dup p.(Asp45fs) pathogenic frameshift mutation	217.6	34.8
100	Male	1961	56	EA	CACNA1A	Heterozygous for the c.2636_2652dup p.(Ala885Thrfs*14) likely pathogenic mutation	82.6	**165.3***
108	Female	1940	77	CANVAS	RFC1	Homozygote for two pathogenic AAGGG repeat expansions of >150 repeats.	34.1	18.3
112	Female	1950	66	CANVAS	RFC1	Homozygote for two pathogenic RFC1 AAGGG repeat expansions of >150 repeats.	**417.4***	26.3
119	Male	1986	31	SCA28	AFG3L2	Heterozygous for c.2069G>T p.(Ser690Ile) variant	76.3	24.2
135	Male	1955	62	Leigh Syndrome	MT-ATP6	Homoplasmic for the m.9176T>C p.(Leu217Pro) pathogenic variant	18.5	20.2
141	Male	1997	21	CACNA1A-related ataxia	CACNA1A	Heterozygous variant for the c.4988G>A; p.Arg1663Gln	21.1	19.7
142	Female	1988	28	SCA26	XRCC1	Homozygous for the c.1293C G p.(Lys43 Asn) likely pathogenic variant	179.5	18.5
148	Male	1990	27	X-linked recessive Charcot-Marie-Tooth disease-4	AIFM1	Hemizygous for the c.784G>A p.(Gly262Ser) likely pathogenic variant c.784G>A p.(Gly262Ser) likely pathogenic variant	91.6	20.2
155	Female	1970	46	FA	FXN	compound heterozygote for two FXN GAA repeat expansions in the pathogenic range.	104.9	**159.4***
156	Male	1978	40	Familial Hemiplegic Migraine type 1, with progressive cerebellar ataxia	CACNA1A	Heterozygous pathogenic mutation c.4999C>T p.(Arg1667Trp)	97.8	43.1

163	Female	1955	63	Occult Macular Dystrophy	RP1L1	Heterozygous for the c.4294_4295insGGCCAGGAGGAGGAAG p.(Ala1432Glyfs*29) likely pathogenic variant	89.4	12.9
166	Male	1980	36	Suggestive (not confirming) of SCA8	SYNE1	Heterozygous for two variants : the likely pathogenic c.15898C>T p.(Arg5300*) and the c.24099+8C>G p.? variant of uncertain significance.	25.3	31.2
172	Female	1981	37	Mast syndrome	SPG21	Homozygous for the c.152_153del p.(Pro51Argfs*38) likely pathogenic variant	87.8	37.5
176	Male	1954	64	EA	CACNA1A	Heterozygous variant for the c.4005delA p.(Gly1336Glufs*36)	41.9	60.1
183	Male	1970	48	SCA3	ATXN3	Heterozygous for one allele with CAG in the expanded pathogenic range	174.1	35.7
184	Female	1956	60	Leigh Syndrome (mitochondrial respiratory chain disease)	MT-ATP	Homoplasmic m.8851T>C p.(Trp109Arg) pathogenic variant	115.7	44.4
191	Female	1965	51	SCA14	PRKCG	Heterozygous for the c.413T>A p.(Val138Glu) likely pathogenic variant	289.4	67.1
193	Male	1975	41	Recessive SCA8	SYNE1	Heterozygous for the c.14077C>T p.(Arg4693*) and c.14287C>T p.(Arg4763*) likely pathogenic variants	142	37
***Values considered elevated**								

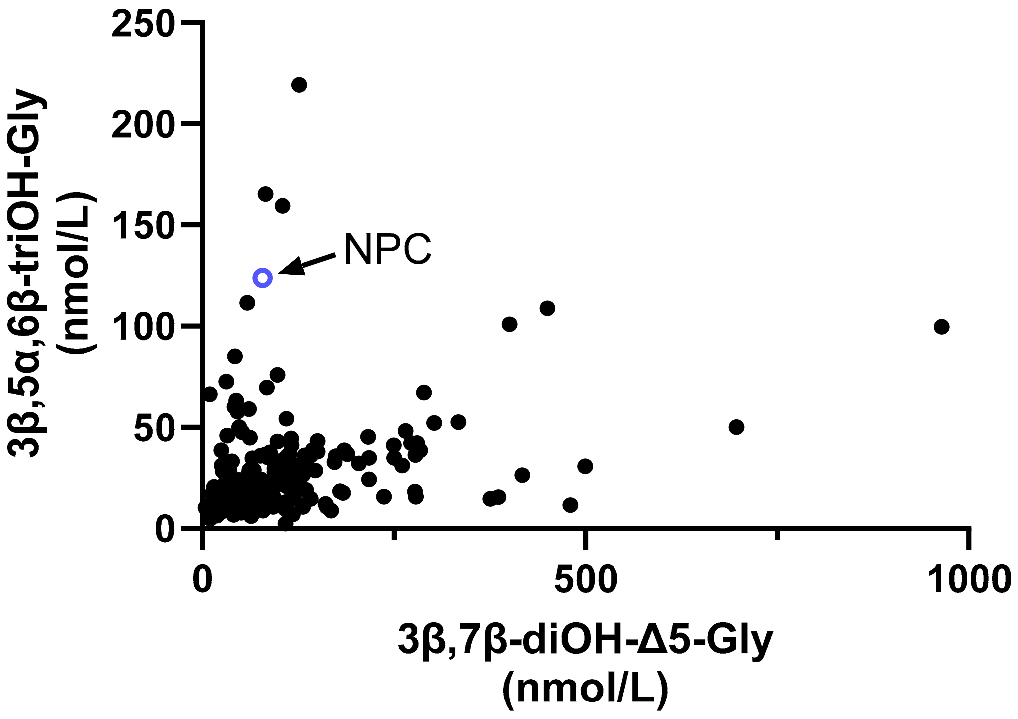

Figure S1: Scatter plot illustrating the relationship between bile acids 3β,5α,6β-triOH-Gly and 3β,7β-diOH-Δ5-Gly, in our study. Each data point on the plot corresponds to a specific patient, with NPC patient being highlighted with an arrow. This patient had 3β,5α,6β-triOH-Gly of 123.9 nmol/L and 3β,7β-diOH-Δ5-Gly of 78.4 nmol/L.

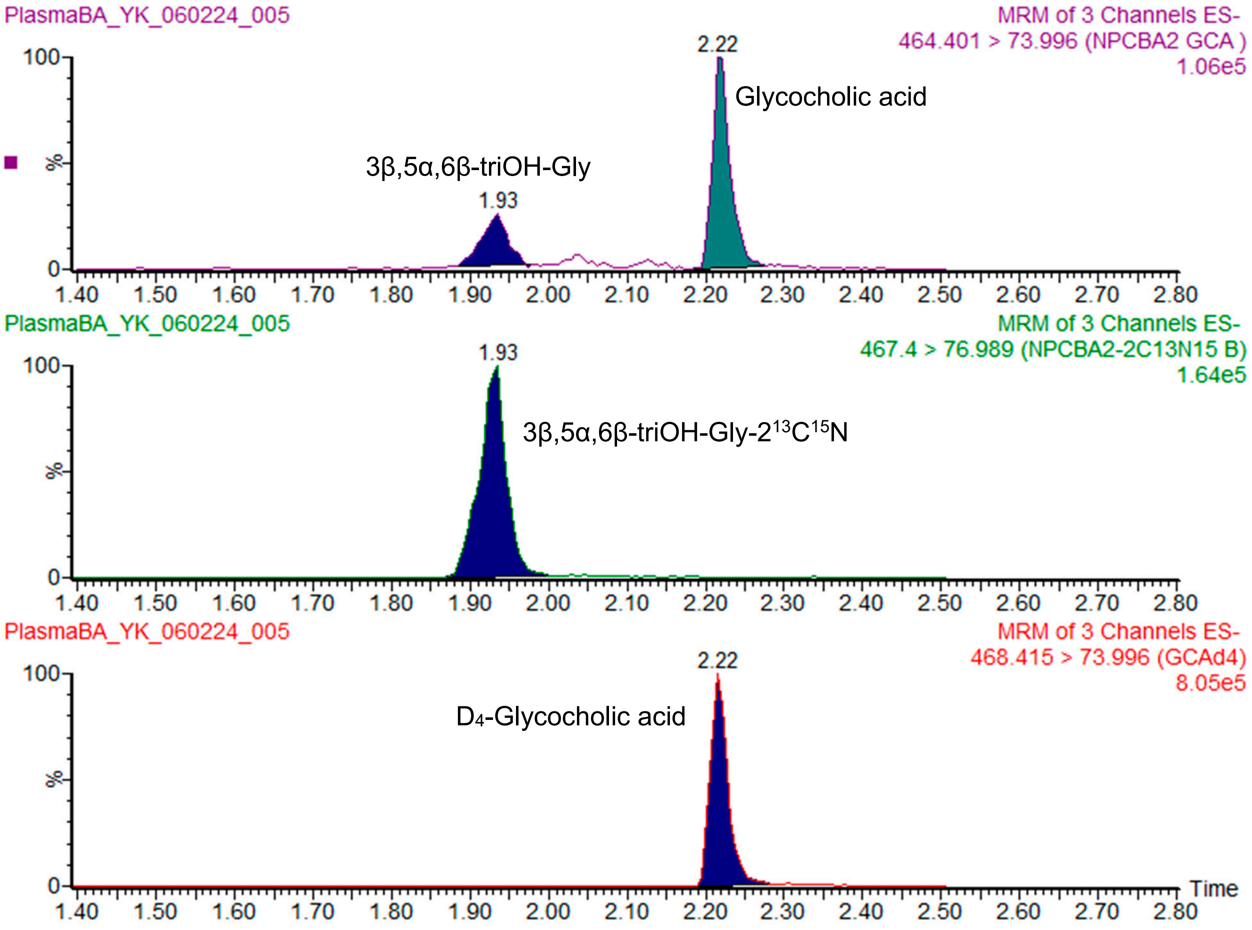

Figure S2: Chromatograms of 3β,5α,6β-trihydroxycholanoyl-glycine and glycocholic acid in plasma from an NPC patient. Identification of 3β,5α,6β-triOH-Gly is confirmed based on its retention time and fragment ion (m/z 464>74).

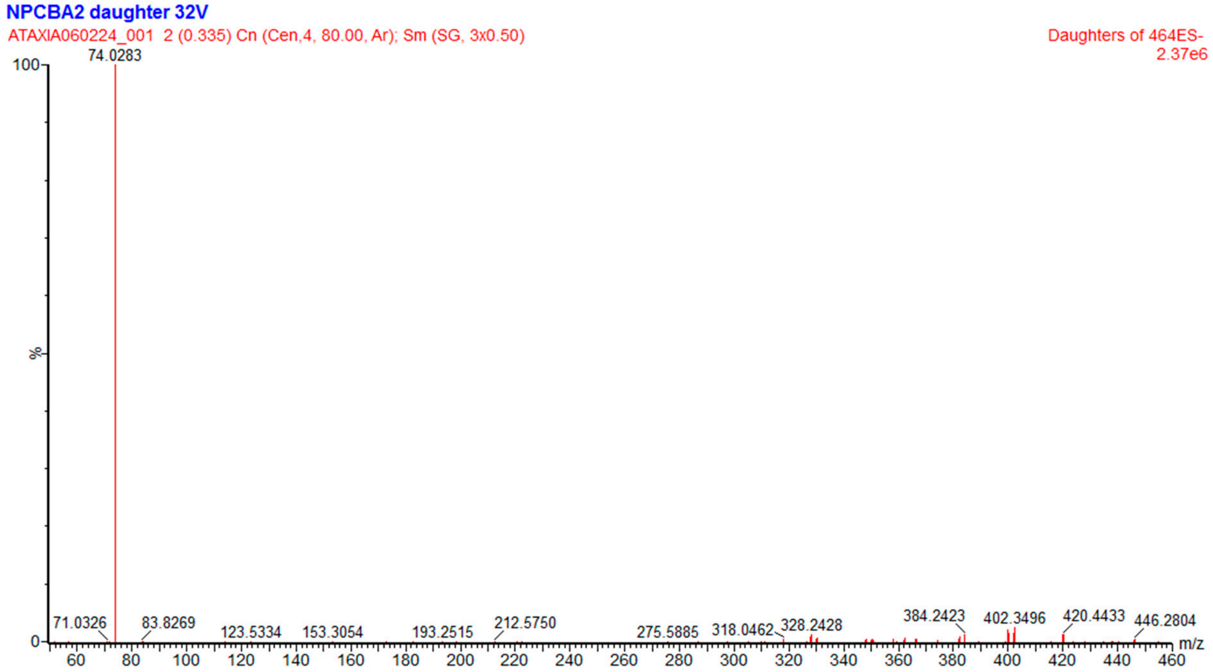

Figure S3: Fragment ions from m/z 464 corresponding to 3β,5α,6β-trihydroxycholanoyl-glycine.
